# Current Notes

**Published:** 1897-03

**Authors:** 


### Current News.

### DENTAL SOCIETY OF THE STATE OF NEW YORK.

The Twenty-ninth Annual Meeting of this society will be held in Albany, May 12 and 13, at which time the following programme will be presented:

President's Annual Address, H. J. Burkhart, D.D.S.

Report of the Correspondent, R. Ottolengui, M.D.S.

Report of the Committee on Practic, A. R. Starr, D.D.S.

"Amalgam Fillings, with a Practical Demonstration," G. V. Black, M.D., D.D.S., Sc.D., Jacksonville, Illinois.

"Dental Organizations," James Truman, D.D.S., Philadelphia.

"Irregularities of the Teeth and their Correction," J. N. Farrar, M.D., D.D.S., New York.

"Cataphoresis," H. W. Gillett, D.M.D., Newport, R. I.

Subject to be announced, B. Holly Smith, M.D., D.D.S., Baltimore.

Members of the profession are fraternally invited to attend.

H. J. Burkhart, D.D.S.,
*President*, Batavia.

C. S. Butler, D.D.S.,
*Secretary*, Buffalo.

---

### TWELFTH INTERNATIONAL MEDICAL CONGRESS.

Moscow, August 19–26, 1897.

### Section of Dentistry.

We have received the following general circular from the members of the committee of this section of the Medical Congress, and give it the publicity that its importance deserves.—[Ed.]

Dear Sir and Colleagues:

The Organizing Committee of the Section of Dentistry of the Twelfth International Medical Congress in Moscow have the honor to send to you herewith the regulation of the congress, together with the programme of the mentioned section, and to respectfully solicit your assistance in insuring the section's success by your presence here, and by a report upon one of the questions in the said programme.

In accordance with the 17th paragraph of the regulation, papers dealing with the subjects named in the programme will have preference over others. This does not, of course, exclude communications upon other subjects, but such communications can only be read provided that time permits. We would therefore venture to suggest that, if possible, the subject of your paper should be chosen from the enclosed list. We feel sure, however, that even should you elect to make a communication upon some question not mentioned in the programme, an opportunity will undoubtedly be found to enable you to read it.

Should you wish to make a report, it is very important to receive the same, or, at any rate, a short account of it, before May 1, 1897, for printing and distribution among the members of the congress.

Trusting to hear favorably from you, we remain, Dear Sir and Colleague,

Yours faithfully,

DR. F. REIN,

*The Manager of the Section.*

DR. I. KOWARSKY,

DR. N. NESMEJANOW,

DR. S. URENIUS,

*The Members of the Committee.*

The address of the Manager: Dr. F. Rein, Moscow, Little Dmitrowka, h. Scheschkow.

THE PROGRAMME OF THE OCCUPATIONS OF THE SECTION OF DENTISTRY OF THE TWELFTH INTERNATIONAL MEDICAL CONGRESS IN MOSCOW.

1. What kind of general and special learning is desirable for the persons who are to occupy themselves with dentistry? The lecturer: *Professor Dr. Julius Scheff* (Vienna).
2. The hygiene of the cavity of the mouth and of the teeth.
3. General and local anæsthetics for tooth-extraction. The lecturer: *Dr. V. Richardson* (London).
4. Cataphoresis in dentistry.
5. The essence and treatment of pyorrhœa alveolaris. The lecturer: *Professor Dr. József Arkövy* (Budapesth).

6. The treatment and filling of the pulpless teeth.

7. Crown- and bridge-work from the hygienic and technical point. The lecturer: *M. Morgenstern* (Baden-Baden).

---

### ODONTOGRAPHIC SOCIETY OF CHICAGO.

At the annual meeting of the Odontographic Society of Chicago, held December 14, 1896, the election of officers resulted as follows: President, Geo. B. Perry; Vice-President, G. W. Schwartz; Treasurer, Edmund Noyes; Secretary, H. H. Wilson.

Member of Board of Directors, W. H. Fox; Board of Censors, E. K. Bennington (chairman), A. G. Johnson, and H. J. Goslee.

H. H. Wilson,
*Secretary.*

---

### THE ST. LOUIS DENTAL SOCIETY—OFFICERS FOR 1897.

President, John H. Kennerly; Vice-President, P. H. Eislœffel; Corresponding Secretary, John G. Harper; Recording Secretary, C. C. Cowderly; Treasurer, A. J. Prosser.

*Committee on Ethics.*—J. E. Pfaff, J. P. Harper, William Conrad.

*Committee on Publication.*—F. F. Fletcher, W. M. Bartlett, De C. Lindsley.

---

### THE CENTRAL DENTAL ASSOCIATION OF NORTHERN NEW JERSEY.

At the Annual Meeting, held on the 15th of February, the following officers were elected for 1897–98: President, Wm. L. Fish, D.D.S., Newark; Vice-President, F. Edsall Riley, D.D.S., Newark; Treasurer, Charles A. Meeker, D.D.S., Newark; Secretary, Herbert S. Sutphen, D.D.S., Newark.

*Executive Committee.*—Geo. E. Adams, D.D.S., South Orange; W. E. Truex, D.D.S., Freehold; C. S. Hardy, D.D.S., Summit; F. S. Gregory, D.D.S., Newark; Fred. C. Barlow, D.D.S., chairman, Jersey City.

H. S. Sutphen, D.D.S.,
*Secretary.*

